# Ceramide metabolism in oxidative and glycolytic muscle: Significance for lipid-induced insulin resistance

**DOI:** 10.1016/j.molmet.2026.102336

**Published:** 2026-02-16

**Authors:** Tova Eurén, Mikael Flockhart, Timotej Strmeň, Xin Zhou, Oscar Horwath, William Apró, Sarah J. Blackwood, Dominik Tischer, Marcus Moberg, Pär Steneberg, Helena Edlund, Abram Katz, Elin Chorell

**Affiliations:** 1 Department of Public Health and Clinical Medicine, Umeå University, Sweden; 2 Department of Medical and Translational Biology, Umeå University, Sweden; 3 Department of Physiology, Nutrition and Biomechanics, Swedish School of Sport and Health Sciences, GIH, Stockholm, Sweden; 4 Department of Women's and Children's Health, Karolinska Institute, Stockholm, Sweden

**Keywords:** Ceramide metabolism, Insulin resistance, Lipidomics, Skeletal muscle fibre, Sphingomyelin synthase 2 (SGMS2)

## Abstract

Altered ceramide accumulation contributes to skeletal muscle insulin resistance, but mechanisms underlying fibre-type-specific susceptibility remain unclear. We hypothesized that fibre-type-specific ceramide metabolism governs vulnerability to lipid-induced insulin resistance. Lipidomics and quantification of ceramide-pathway enzymes were performed in mouse skeletal muscles with distinct fibre-type composition (oxidative, mixed and glycolytic) from control-diet (n = 12) and high-fat-diet (HFD; n = 12) mice. In humans, lipidomics and enzyme profiling were done in vastus lateralis biopsies from 36 adults stratified into oxidative or glycolytic phenotypes; insulin sensitivity was determined by glucose tolerance testing. siRNA-mediated silencing of SGMS1 and SGMS2 followed by lipidomics probed sphingomyelin–ceramide cycling in human myoblasts. In mouse muscle, ceramide composition rather than total content, differed by fibre type: oxidative muscle was enriched in very-long-chain ceramides, whereas glycolytic and mixed muscles contained higher C18-ceramides, paralleled by fibre-type-specific expression of enzymes involved in *de novo* synthesis and sphingomyelin–ceramide cycling. HFD induced ceramide remodelling, with C18-ceramides accumulating in oxidative and mixed muscles and very-long-chain species decreasing in glycolytic muscle; among all assessed enzymes, only SGMS2 was significantly downregulated in oxidative muscle. In humans, an oxidative phenotype associated with higher very-long-chain ceramides and insulin sensitivity, whereas a glycolytic phenotype displayed higher C16–18 ceramides, higher SGMS1 and SMPD2 expression, and lower insulin sensitivity. Elastic net regression identified C16–18 ceramides and galactosylceramides as negative predictors of insulin sensitivity. SGMS2 silencing caused broader ceramide accumulation than SGMS1 silencing, supporting a central role for SGMS2-mediated sphingomyelin–ceramide cycling in limiting ceramide burden.

## Highlights

• C18-ceramides accumulate in glycolytic muscle linked to insulin resistance.
• SGMS2 downregulation in oxidative muscle impairs ceramide clearance.
• SGMS2 silencing increases ceramides in human muscle cells.
• Muscle C18-ceramides predict lower insulin sensitivity in humans.
• Muscle fibre type influences lipid metabolism and insulin action.

## Graphical abstract

**Figure:**
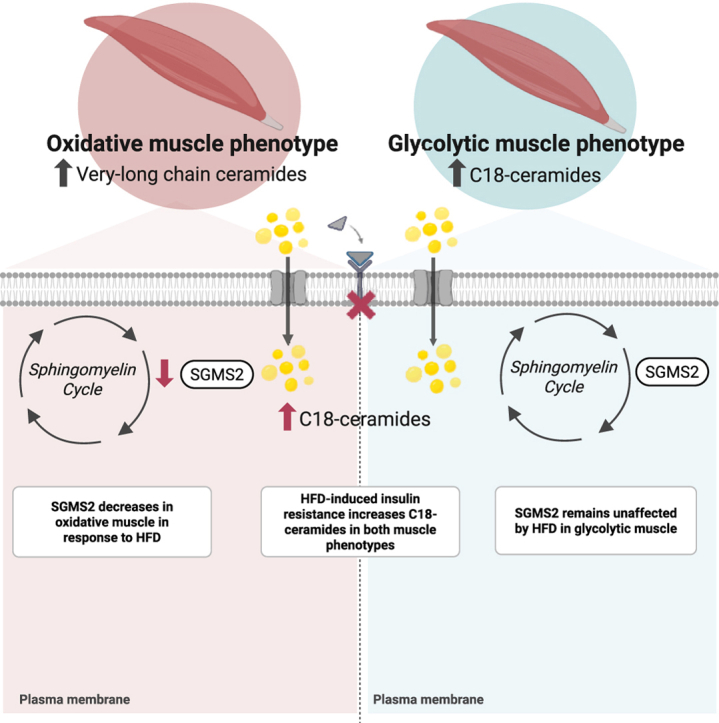

## 1 Introduction

Lipid accumulation in skeletal muscle is frequently observed in insulin resistance [1], yet not all lipid accrual is deleterious: endurance trained athletes exhibit high intramyocellular lipid stores while maintaining insulin sensitivity [2]. Among these lipids, ceramides, a subclass of sphingolipids, can act as bioactive messengers that impair insulin signaling [3], trigger stress pathways such as JNK and PKC ζ–PP2A [4], and disrupt membrane organisation [5].

Considerable experimental evidence indicates that *de novo* ceramide synthesis contributes to insulin resistance. In rodents, inhibition of serine palmitoyltransferase (SPTLC), the rate-limiting enzyme of *de novo* ceramide synthesis pathway, lowers muscle ceramide content and reverses diet-induced insulin resistance [6]. Similarly, genetic loss of ceramide synthase (CerS) 1, which preferentially generates C18-ceramides, has been shown to improve systemic glucose metabolism [7].

However, this *de novo*-centric model is not fully validated in human skeletal muscle in vivo. Transcriptional signatures of *de novo* ceramide synthesis are heterogeneous, with CerS3/CerS6 upregulated in insulin resistant muscle in one cohort, no differences in SPTLC isoforms or other ceramide-metabolism transcripts in others, and increased expression of sphingolipid-synthesis genes in highly insulin-sensitive athletes [[8], [9], [10]]. Together, these observations question the extent to which increased *de novo* synthesis alone drives ceramide accumulation in vivo and suggest that additional pathways, fibre-type-dependent flux, post-transcriptional regulation, and compartmentalized turnover, may contribute to the selective accumulation of ceramide species linked to impaired insulin sensitivity [11].

Skeletal muscle consists of oxidative (type I) and glycolytic (type II) fibres, and their divergent metabolic programs shape both lipid handling and whole-body insulin sensitivity [12,13]. This cellular heterogeneity has been largely overlooked in human studies, yet it may critically modulate how ceramides accumulate and act within muscle. Type I fibres are rich in mitochondria and insulin-signaling proteins [14], are primed towards oxidative metabolism, whereas type II fibres have greater glycolytic capacity [15]. Consistently, a low proportion of type I fibres correlates with reduced insulin sensitivity [16], and we recently showed that ceramide accumulation is associated with fibre-type composition and insulin sensitivity in humans [17].

Adding further complexity, ceramides form a diverse class of lipids that vary in sphingoid backbone and acyl-chain composition, features that determine their biological activity. Long-chain ceramides (C16–C18) are consistently associated with metabolic dysfunction [7,18,19], while very-long-chain ceramides (>C20) are less explored but appear to serve structural barrier-forming or regulatory signaling roles [20]. We therefore hypothesize that both the chemical composition of ceramide species and their regulation vary with muscle fibre-type composition. Adopting a fibre-type-specific perspective may thus help to resolve conflicting results and clarify mechanisms of lipid-induced insulin resistance.

## 2 Material and methods

### 2.1 Animal model and experimental design

Male CBA/CaCrl mice (Charles River, UK) were crossed with female C57BL/6J mice (Jackson Laboratory, US), and male F1 offspring were used. These strains are prone to diet-induced obesity and insulin resistance, making them suitable models for studies of metabolically induced insulin resistance. Additionally, the use of F1 mice obtained from breeding male C57BL/6 J mice with female CBA/CaCrl was aimed at avoiding potential strain-specific effects related to inbreeding, thereby better mimicking the genetic diversity of natural populations.

Mice were housed at 22 °C, 50% humidity, with a 12:12 h light/dark cycle. At 10 week of age, F1 littermates were randomly assigned to a high-fat diet (HFD, n = 12; D12492 Research Diets, Inc.) or control diet (CON, n = 12; D12450J, Research Diets, Inc.) for nine weeks (sufficient to induce insulin [21], with food and water ad libitum. All procedures complied with the Guidelines for the Care and Use of Laboratory Animals and were approved by the regional Animal ethics Committee (A4-19).

### 2.2 Metabolic characterization of mouse muscle and insulin sensitivity

Plasma insulin levels were measured with an ultrasensitive ELISA (Chrystal Chem Inc., #90080), and blood glucose were determined with a glucometer (Accu-Chek Aviva, Roche, Sweden). Insulin resistance was estimated by HOMA-IR, calculated as fasting glucose (mM) x fasting insulin (μU/ml)/22.5. An intraperitoneal glucose tolerance (IPGTT) test was performed to assess glucose and insulin dynamics in the mouse model. IPGTT were conducted at baseline and after 9 weeks HFD or CON feeding. Following a 16 h fast mice were anesthetized with a mixture of 1:4 Hypnorm (Veta Pharma) and 1:4 Midazolam (Mercury Pharmaceuticals) before intraperitoneal glucose injection (2 mg/g; Gibco). Blood glucose levels were monitored from the tail vein at baseline and at 10, 30, 60, 90, and 120 min after glucose administration. Plasma insulin concentrations were determined in blood plasma collected at baseline and at 2.5, 5, 10, 30, and 60 min after glucose injection. Body weight was recorded weekly, and body composition assessed by EchoMRI (EchoMRI LLC; EchoMRI 3-in-1) after 9 weeks on diet (age 19 weeks). Vastus lateralis, extensor digitorum longus (EDL), and soleus muscles were excised, cleaned of connective tissue, snap-frozen in liquid nitrogen and stored at −80 °C. These muscles were selected for their distinct fiber-type composition: oxidative soleus (∼40% type I, ∼50% type IIa, ∼10% type IIx fibers, and minimal type IIb) [22]; highly glycolytic vastus lateralis, (>95% type IIx/IIb fibers, negligible type I) [23]; and EDL with intermediate profile (∼66–73% type IIb and 21–26% type IIx) [24,25].

### 2.3 Human participants and study design

The study was approved by the Swedish Ethical Review Authority (Dnr 2021–03964). Participants were screened with a general medical status form, including training habits, and only healthy individuals were included. Most were recreationally active but not highly trained or competing athletes. Exclusion criteria were obesity and having a first-degree with diabetes. Participant characteristics have been reported in detail elsewhere [26]. The final cohort comprised 36 participants (22 females and 14 males; 26.5 ± 5.8 years). Muscle fibre type composition was determined via electrophoretic separation of myosin heavy chain isoforms and immunohistochemistry [27]. Participants were a priori divided into two groups based on type I fibre content in vastus lateralis: type I-dominant (OX: >50% type I fibres) and type II-dominant (GLY: <40% type I fibres).

### 2.4 Human clinical and biochemical assessments

Whole-body insulin sensitivity (SIgalvin) [28] was established via an intravenous glucose tolerance test (IVGTT). Glucose (300 mg/mL; Apl, Stockholm, Sweden) was infused continuously for 2 min (0.3 g/kg body weight), and blood samples were collected over 90 min. Muscle biopsies were taken from the vastus lateralis after an overnight fast using the Bergström needle technique with suction under local anesthesia. Biopsies were cleaned of non-muscle constituents, snap-frozen in liquid nitrogen, and stored at −80 °C. All analyses were performed on freeze-dried tissue.

### 2.5 siRNA-mediated silencing of SGMS1 and SGMS2 in human primary myoblast

Human primary myoblast cultures were established from residual tissues of m. semitendinosus obtained during Anterior Cruciate Ligament reconstruction surgery, washed in HBSS, and cultured as previously described [29]. Cells (1.5 × 10ˆ5 per well) were incubated overnight (37 °C, 5% CO_2_) and characterized by Pax7, Myf5, MyoD, MyoG, Myh1, and Myh2. For RNAi, 25 pmol of SGMS1 (Invitrogen, 149 155) or SGMS2 (Invitrogen, 128 040) siRNA was complexed with 6 μL Lipofectamine RNAiMAX in 125 μL Opti-MEM® I Reduced Serum Medium, incubated 5 min at room temperature, and 250 μL was added per well (antibiotic-free). Knockdown was verified 48 h later by Western blot analysis. Antibodies used are listed in Supplementary Table S1. The study protocol was approved by the Swedish Ethical Review Authority (dnr 2021-05627-01).

### 2.6 Mass spectrometry-based analyses

Mouse and human skeletal muscle lipids and mouse polar metabolites were analysed by targeted LC-TOF/MS (Agilent Technologies Inc.) as described previously [30]. Briefly, lipids were extracted using a biphasic protocol [31] and polar metabolites, including anserine, with a dual extraction method [32]. Samples were randomized to balance groups and minimize analytical biases. Data were processed in Agilent MassHunter Profinder (version B.08.00), and lipids annotated according Lipid Maps nomenclature (lipidmaps.org). Metabolites were identified by MS/MS and retention times of standards. Metabolite levels were normalized using a labelled internal standard (DG(18:0/0:0/18:0)-d_5_, Avanti Polar Lipids, #800855P) at known concentrations.

### 2.7 Tissue homogenization and immunoblotting

Mouse soleus, EDL and vastus lateralis (15–30 mg, wet) and human vastus lateralis (∼3 mg, dry) were homogenized (25 μL buffer mg^−1^ wet muscle; 100 μL mg^−1^ dry muscle) in buffer (2 mM HEPES pH 7.4, 1 mM EDTA, 5 mM EGTA, 10 mM MgCl_2_, 50 mM β-glycerophosphate, 1% Triton X-100, 2 mM DTT, 1% phosphatase inhibitor, 1% Halt Protease Inhibitor). Lysates were centrifuged (10 000×*g*, 10 min, 4 °C), and the supernatant was transferred to new tubes. The protein concentration was measured (Pierce 660 nm assay; Thermo Fischer Scientific), diluted to 1 mg/mL with 4 × Laemmli/2- Mercaptoethanol (90:10), denatured (95 °C, 5min), and stored at −80 °C.

10 μg of protein was run on TGX Stain-Free™ gels (Bio-Rad), transferred to PVDF membranes (Trans-Blot Turbo, Bio-Rad), and transfer efficiency verified by Pierce™ Reversible Protein Stain (Thermo), and total protein used as loading control. Membranes were blocked (1 h, RT; 5% non-fat dry milk/TBS-T) and incubated overnight (4 °C) in TBS with 2.5% milk and 1% Tween-20. Antibodies are listed in Supplementary Table S1.

### 2.8 Statistical analyses

All analyses were performed using R statistical software (v4.3.1; R Core Team 2023) [33]. Normality was assessed (Shapiro–Wilk), and appropriate statistical tests were applied. Initial Principal Component Analysis (PCA) identified one HFD vastus sample as an outlier which was excluded due to low analytical quality (n = 12 → 11). There were no human outliers. Orthogonal Projections to Latent Structures Discriminant Analysis (OPLS-DA) was conducted (ropls package [34]). Model robustness and reliability were assessed through permutation testing (500 iterations) and discriminatory lipids met variable importance in projection (VIP) > 1.0, |Cliff's δ| > 0.5, and p < 0.05 (two-sided Wilcoxon rank-sum).

Lipids predicting insulin sensitivity were selected by elastic net regression (glmnet package [35]; α = 0.8, λ = λ.min, 10-fold CV, 100 bootstrap resamples). Key lipid predictors were identified by non-zero coefficients at λ.min and model performance was assessed by the mean and standard deviation of the mean squared error.

Enzyme and lipid concentrations (log_2_) were analysed with a type-II two-way ANOVA (Muscle x Treatment), car package [36], with significant terms (p < 0.05) further assessed by two-sided pairwise t-tests.

## 3 Results

### 3.1 Muscle phenotypes dictate lipid profiles

**Figure 1:**
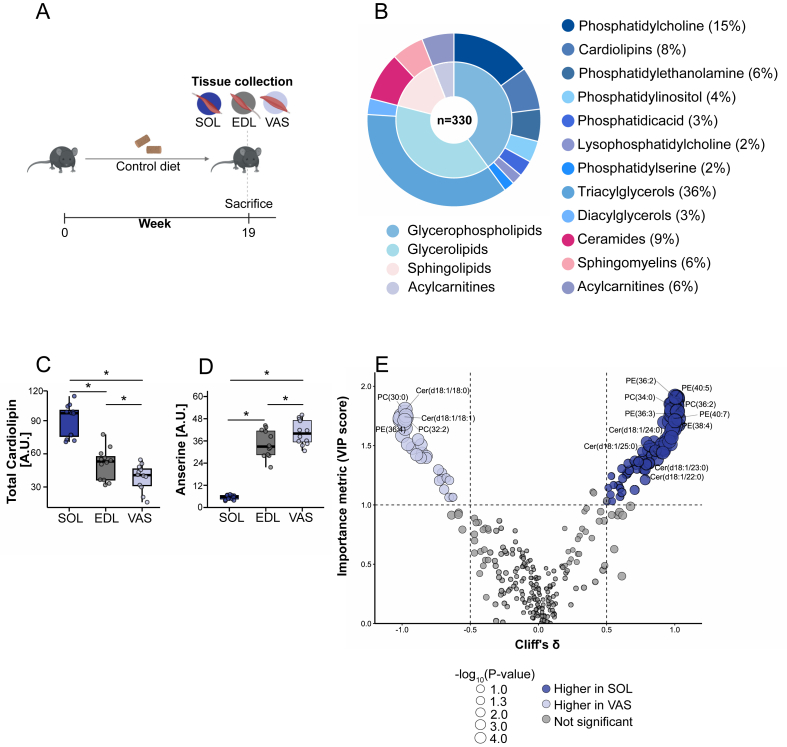
**Comparative analysis of skeletal muscle lipid composition and metabolic markers in mice**. (a) Schematic overview of the study design, showing tissue collection from three hindlimb muscles: soleus (SOL), extensor digitorum longus (EDL), and vastus lateralis (VAS) after 9 weeks of control diet feeding (n = 12 per muscle group). (b) Sunburst plot displaying the distribution of 330 detected lipid species, grouped into major lipid classes across all muscle samples. (c) Total cardiolipin content in SOL, EDL, and VAS, to characterize their mitochondrial content. (d) Anserine content in SOL, EDL, and VAS muscles, reflecting their glycolytic profile. (e) Volcano plot comparing SOL vs. VAS, showing effect size (Cliff's δ, x-axis) and variable importance (VIP score, y-axis) from an OPLS-DA model. Dot size corresponds to univariate significance (−log10 p-value, Wilcoxon rank-sum). Lipids meeting pre-defined thresholds (VIP >1, |Cliff's δ| > 0.5, −log10(p) > 1.3) are highlighted, with light blue indicating enrichment in VAS and dark blue enrichment in SOL. Box plots show median, IQR, and ±1.5 × IQR whiskers, with individual data points overlaid. Log_2_-transformed values were analysed by two-way ANOVA with pairwise two-sided Student's t-tests. Significant differences are indicated by p < 0.05. Abbreviations: SOL, soleus; EDL, extensor digitorum longus; VAS, vastus lateralis.

Comprehensive lipidomics profiling was performed on mouse soleus (oxidative), EDL (mixed), and vastus lateralis (glycolytic), identifying 330 lipids spanning 12 lipid classes and four major categories, including sphingolipids, acylcarnitines, glycerolipids, and glycerophospholipids (Figure 1A–B). To confirm distinct metabolic phenotypes, we quantified cardiolipin, a marker of mitochondrial content and oxidative capacity [37], and anserine, a methylated derivative of carnosine associated with glycolytic metabolism [38]. Metabolite levels were normalized using a labelled internal standard (DG(18:0/0:0/18:0)-d_5_, Avanti Polar Lipids, #800855P) at known concentrations.

Cardiolipin levels were ∼twofold higher in soleus than in EDL and vastus lateralis (Fig. 1C), whereas anserine levels were ∼6-8-fold higher in EDL and vastus lateralis than in soleus (Fig. 1D), validating their oxidative and glycolytic properties, respectively. EDL displayed intermediate levels of both, consistent with its mixed fibre-type profile.

**Figure 2:**
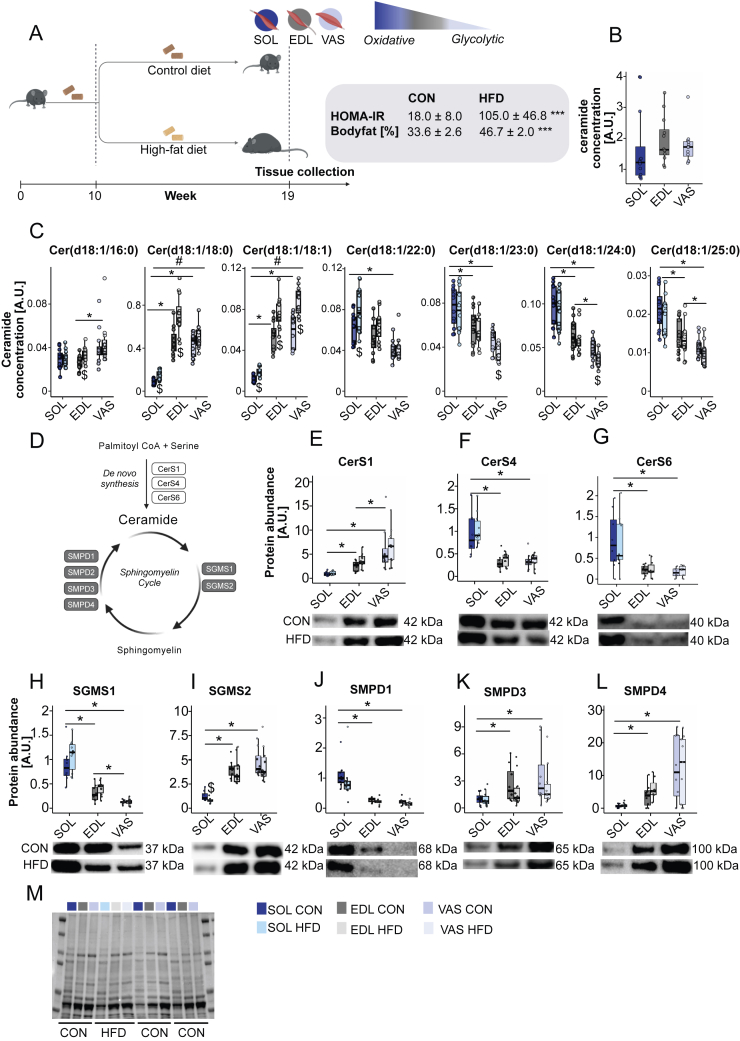
**Ceramide metabolism and acyl-chain distribution across oxidative and glycolytic mouse skeletal muscles**. (a) Schematic of the diet intervention, in which mice were fed a control diet (CON; n = 12) or a high-fat diet (HFD; n = 12) for 9 weeks, after which SOL, EDL, and VAS muscles were collected. (b) Total ceramide concentrations in SOL, EDL, and VAS (n = 12). (c) Ceramide acyl-chain distribution across SOL, EDL, and VAS, stratified by diet (n = 12). (d) Overview of ceramide metabolic pathways, illustrating *de novo* synthesis and the sphingomyelin cycle, in which SMPD1/SMPD3/SMPD4 generate ceramide via sphingomyelin hydrolysis, and SGMS1/SGMS2 regenerate sphingomyelin. (e–g) Protein abundance of CerS1, CerS4, and CerS6 in SOL, EDL, and VAS, with representative Western blots shown (CerS1/CerS4: n = 8 CON, n = 10 HFD; CerS6: n = 7 CON, n = 9 HFD). (h–i) Protein abundance of SGMS1 and SGMS2 in SOL, EDL, and VAS, with representative Western blots shown (n = 8 CON, n = 10 HFD). (j–l) Protein abundance of SMPD1, SMPD3, and SMPD4 in SOL, EDL, and VAS, with representative Western blots shown (n = 8 CON, n = 10 HFD). (m) MemCode staining used for total-protein normalization. Box plots show median, IQR, and ±1.5 × IQR whiskers with individual points overlaid. Ceramide concentrations were normalized using a stable labelled internal standard. Log_2_-transformed values were analysed by two-way ANOVA (muscle × treatment), followed by post hoc pairwise two-sided Student's t-tests. Significance symbols: # interaction effect; ∗ pairwise muscle comparisons; $ pairwise treatment comparisons; statistical significance set at p < 0.05. Abbreviations: SOL, soleus; EDL, extensor digitorum longus; VAS, vastus lateralis. All three muscles were analysed from each mouse included in this study, and representative immunoblots for each protein were obtained from the same membrane.

Although ceramides accounted for only ∼9% of the total muscle lipidome (Fig. 1B), specific ceramide species emerged as key discriminators between oxidative and glycolytic muscles. Total ceramide abundance did not differ significantly between muscles (Figure 2B), suggesting that composition rather than content underlies phenotype-specific effects.

To identify lipid signatures discriminating oxidative from glycolytic phenotypes, we compared the lipidomes of the two most divergent muscles, soleus and vastus lateralis, using OPLS-DA. The model showed strong predictive performance (Q^2^ = 0.953), and permutation testing confirmed its robustness (pQ^2^ = 0.002).

We observed pronounced differences in long- and very-long-chain ceramides and specific phospholipids. Soleus showed higher levels of very long-chain ceramides [Cer(d18:1/25:0), Cer(d18:1/24:0), Cer(d18:1/23:0), and Cer(d18:1/22:0)] and phospholipids [PE(40:5), PE(36:2), PC(36:2), PC(34:0), PE(40:7), PE(36:3), and PE(38:4)], whereas vastus lateralis had higher long-chain ceramides [Cer(d18:1/18:0) and Cer(d18:1/18:1)] and phospholipids such as PC(30:0), PE(36:4), and PC(32:2) (Fig. 1E). Across all three muscles, acyl-chain-specific analysis revealed a consistent pattern: oxidative soleus contained significantly lower C18-ceramides [Cer(d18:1/18:0) and Cer(d18:1/18:1)] and higher levels very long-chain C22–C25-ceramides [Cer(d18:1/23:0), Cer(d18:1/24:0) and Cer(d18:1/25:0)] than both EDL and vastus lateralis (Fig. 2C), indicating that muscle phenotype dictates ceramide composition.

### 3.2 Muscle phenotype-specific regulation of ceramide metabolism

To investigate mechanisms underlying fibre-type-specific differences in ceramide composition, we assessed protein abundance of endoplasmic-reticulum-resident ceramide synthase (CerS) isoforms (Fig. 2D). Immunoblotting revealed muscle phenotype-specific expression. CerS1 was lowest in oxidative soleus and markedly higher in glycolytic vastus lateralis and mixed-type EDL (Fig. 2E). In contrast, CerS4 was enriched in soleus relative to both EDL and vastus lateralis (Fig. 2F). These patterns broadly mirrored the ceramide profiles, with higher C18-ceramides in glycolytic/intermediate muscles (vastus lateralis, EDL) and elevated very-long-chain ceramides in oxidative soleus, suggesting that ceramide acyl-chain composition is partly determined by fibre-type-specific CerS expression. Importantly, however, CerS4 exhibits context-dependent acyl specificity, and its apparent product profile can vary with substrate (acyl-CoA) availability and cellular context [39]. Thus, the association between higher CerS4 abundance and very-long-chain ceramides likely reflects a combination of CerS4 expression and fibre-type-specific differences in lipid substrate supply and flux.

We therefore expanded the analysis to include CerS6, an isoform linked to long-chain ceramide synthesis. In mice, CerS6 protein was significantly higher in oxidative soleus compared with both mixed (EDL) and glycolytic (vastus lateralis) muscles (Fig. 2G). Given CerS6's known preference for generating C16:0–ceramide [40], this result would predict higher C16:0-ceramide levels in oxidative muscle. However, targeted lipidomics showed the opposite pattern, with glycolytic vastus lateralis exhibiting the highest C16:0-ceramide content (Fig. 2C). This mismatch between enzyme expression and ceramide accumulation suggests that fibre-type differences in C16:0-ceramide content cannot be explained by *de novo* synthesis alone, and instead implicates downstream regulatory processes, including sphingomyelin–ceramide cycling.

To examine sphingomyelin-ceramide cycling (Fig. 2D), we quantified ceramide-clearing enzymes (SGMS1, SGMS2) and ceramide-producing enzymes (SMPD1, SMPD3, SMPD4). SGMS1, a sphingomyelin synthase localised to the ER/Golgi [41], was highest in oxidative soleus, intermediate in EDL, and lowest in glycolytic vastus lateralis (Fig. 2H). SGMS2, which resides at the plasma membrane [41], showed the reciprocal pattern (Fig. 2I). Among ceramide-producing enzymes, SMPD1 was most abundant in soleus (Fig. 2J), whereas SMPD3 and SMPD4 were higher in EDL and vastus lateralis (Fig. 2K-l). Together, these data indicate that fibre-type-specific ceramide profiles reflect orchestrated by distinct programs involving both *de novo* ceramide synthesis and compartmentalized ceramide clearance via sphingomyelin-ceramide cycling.

High-fat diet drives muscle-specific ceramide remodelling linked to insulin resistance.

To investigate how lipid-induced insulin resistance influences ceramide metabolism across muscles, we compared mice fed a high-fat diet (HFD, n = 12) with control littermates on standard diet (CON, n = 12). As expected, HFD feeding caused a marked increase in body fat [33.6 ± 2.6% (CON) vs. 46.7 ± 2.0% (HFD)] and systemic insulin resistance, reflected by higher HOMA-IR [18.0 ± 8.0 (CON) vs. 105.0 ± 46.8 (HFD)] (Fig. 2A). Diet induced insulin resistance was also confirmed by glucose tolerance and glucose-stimulated insulin secretion tests where there was no difference between groups prior to diet initiation, whereas after 9 weeks HFD mice displayed impaired glucose tolerance (higher glucose at 0, 30, 60 and 90 min during an intraperitoneal glucose tolerance test) and increased insulin secretion (higher insulin at 30 and 60 min during an intraperitoneal glucose-stimulated insulin secretion test) compared with CON (n = 7/group; Supplementary Fig. 5).

Ceramide remodelling in response to HFD was muscle specific. Oxidative soleus and intermediate EDL accumulated long-chain ceramides, including Cer(d18:1/18:0) and Cer(d18:1/18:1), whereas glycolytic vastus lateralis showed selective reductions in very-long-chain ceramides, Cer(d18:1/23:0) and Cer(d18:1/24:0), which remained unchanged in soleus and EDL (Figure 2C and m). Notably, HFD also selectively increased C16:0-ceramide levels in the mixed EDL muscle, with no corresponding increase in soleus or vastus lateralis. These findings suggest that oxidative and glycolytic muscles engage distinct ceramide regulatory mechanisms under lipid-induced insulin resistance.

To explore underlying mechanisms, we assessed ceramide-regulating enzymes after HFD, including *de novo* synthesis (CerS1, Cers4) and sphingomyelin-ceramide cycling (SGMS1, SGMS2, SMPD1, SMPD3, SMPD4). Among these, only SGMS2, a plasma membrane-associated ceramide-clearing enzyme, was significantly downregulated, selectively in oxidative soleus (Fig. 2I), while all others were unchanged (Figure 2E–H, 2j-l, and 2m). Together, these data indicate that HFD induces muscle-type-specific alterations in ceramide metabolism, with oxidative muscle particularly prone to ceramide accumulation due to selective SGMS2 suppression, supporting a model in which impaired ceramide clearance rather than biosynthesis is a key node linking muscle phenotype to lipid-induced insulin resistance.

### 3.3 Compartment-specific ceramide clearance drives ceramide accumulation in human-derived myoblasts

**Figure 3:**
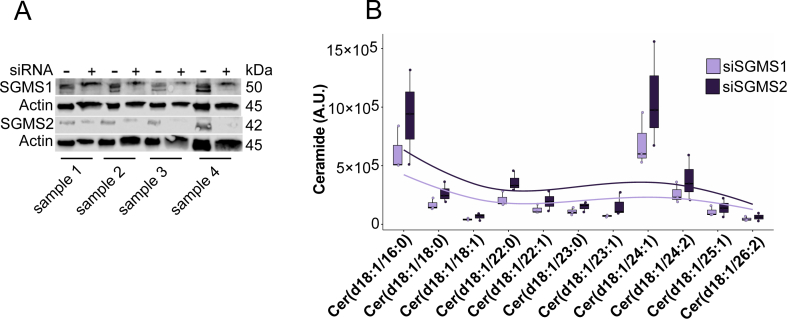
**SGMS1/2 knockdown alters ceramide levels in human myoblasts**. (a) Representative Western blot validating siRNA-mediated knockdown of SGMS1 and SGMS2 in human myoblasts (n = 3). Actin is shown as a loading control. (b) Ceramide species distribution following SGMS1 (light purple) or SGMS2 (dark purple) knockdown. Box plots show the median, IQR, and ±1.5 × IQR whiskers with individual points overlaid and trendlines to illustrate overall shifts in ceramide composition.

To further dissect the contributions of SGMS isoforms in intracellular ceramide accumulation, we used an in vitro model of human skeletal muscle and selectively silenced SGMS1 and SGMS2 in primary human myoblasts using siRNA. This allowed us to assess their distinct roles in regulating ceramide levels under controlled conditions. Knockdown efficacy was confirmed by immunoblotting, showing a significant and comparable reduction in SGMS1 and SGMS2 protein levels across all donors (Figure 3A). Lipidomic profiling revealed that SGMS2 silencing caused a broader and more pronounced accumulation of ceramide species than SGMS1 knockdown (Fig. 3B). The increase was uniform across ceramide chain lengths, with no enrichment of specific acyl-chain species, suggesting that SGMS2 plays a dominant role in maintaining overall ceramide homeostasis in human myoblasts. Although statistical power was limited by sample size, this pattern was consistent across all donors, supporting SGMS2 as a primary regulator of intracellular ceramide burden.

Notably, C16-ceramide, together with C24:1-ceramide, emerged as the most abundant ceramide species in cultured human myoblasts, contrasting with the C18-ceramide dominance observed in vivo in skeletal muscle. This discrepancy is consistent with reports that C16-ceramide promotes insulin resistance in cultured myocytes [42] and highlights limitations of in vitro systems for modelling ceramide metabolism in vivo. These findings underscore the need for cautious interpretation of cell-based models and for validating observations in physiologically relevant systems.

### 3.4 Sphingomyelin-ceramide cycle intermediates mirror muscle-specific lipid alterations

Both DAGs and SMs are linked to sphingolipid and glycerolipids metabolism and have established links to insulin resistance [43]. DAG accumulation in skeletal muscle is known to vary between fibre types and has been implicated in impaired insulin signaling [17]. We therefore examined the distribution of SM and DAG species across mouse muscle types, and in response to HFD.

Skeletal muscle SM composition largely mirrored ceramide patterns. Oxidative soleus accumulated long-chain SM species such as SM(d18:1/18:0) with HFD, whereas very-long-chain SMs, including SM(d18:1/24:0), SM(d18:1/24:1), and SM(d18:1/24:2), were selectively reduced in glycolytic vastus lateralis (Supplementary Fig. 1). DAG species also showed muscle-specific patterns: EDL contained the highest levels of several saturated and polyunsaturated species, including DAG(32:0), DAG(36:4), and DAG(40:7), whereas vastus lateralis showed lower or intermediate levels. Soleus was uniquely enriched in DAG(36:3). HFD increased most DAG species in soleus and EDL, with smaller effects in vastus lateralis (Supplementary Fig. 2).

### 3.5 Human muscle phenotype influences ceramide metabolism and lipid-induced insulin resistance

**Figure 4:**
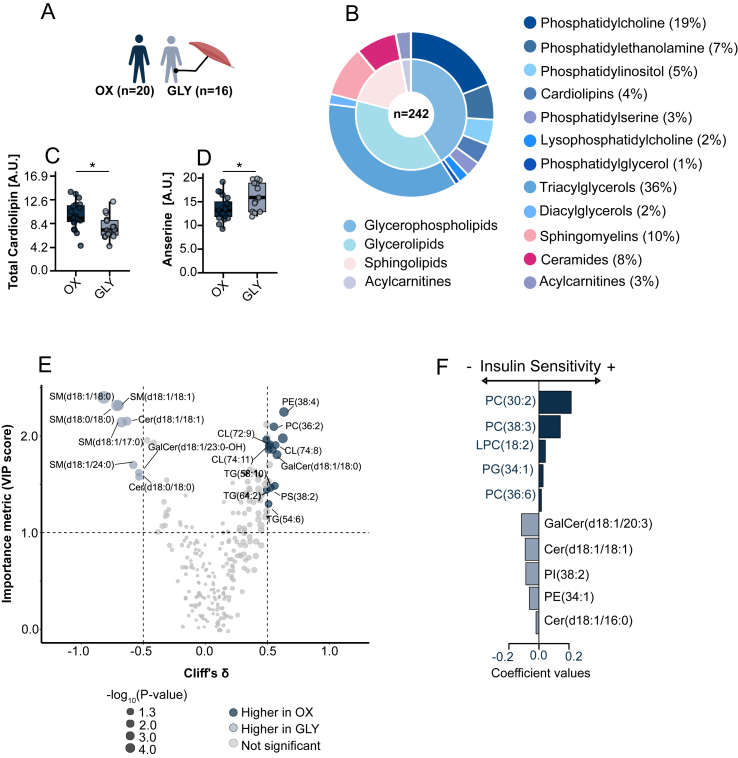
**Lipidomic differences between human type I (OX) and type II-dominant (GLY) muscle phenotypes and identification of lipids predictors of insulin sensitivity**. (a) Overview of participant stratification, showing healthy recreationally active subjects expressing dominance of myosin heavy chain type I- (>50% type I, n = 20) or type II (<40% type I, n = 16) in the vastus lateralis muscle. (b) Sunburst plot displaying the distribution of 242 detected lipid species, grouped into major lipid classes across OX and GLY muscle samples. (c–d) Total cardiolipin and anserine content in OX (n = 20) versus GLY (n = 16) muscle, reflecting differences in mitochondrial and glycolytic muscle phenotype, respectively. (e) Volcano plot comparing OX versus GLY muscle, showing effect size (Cliff's δ, x-axis) and variable importance (VIP score, y-axis) from an OPLS-DA model. Dot size corresponds to univariate significance (−log10 p-value). Lipids meeting VIP >1, |Cliff's δ| > 0.5, and p < 0.05 are highlighted, with grey-blue enriched in GLY, navy enriched in OX, and grey indicating non-significant species. (f) Elastic net regression coefficients for lipid predictors of insulin sensitivity. Navy bars denote lipids enriched in OX muscle, while grey-blue bars denote lipids enriched in GLY muscle; positive coefficients represent positive associations with insulin sensitivity. Box plots show the median, IQR, and ±1.5 × IQR whiskers with individual points overlaid. Between-group comparisons were performed using two-sided unpaired Student's t-tests (p < 0.05).

Biopsies from oxidative (OX, n = 20) and glycolytic (GLY, n = 16) participants were subjected to lipidomics profiling (Figure 4A). The OX group exhibited significantly higher insulin sensitivity than GLY, as assessed by the SIgalvin index (Table 1). Baseline characteristics and immunohistochemistry for this cohort have been reported previously [26].

**Table 1:** Participant characteristics and metabolic parameters in groups with predominantly oxidative (OX) or glycolytic (GLY) muscle phenotypes.

Variable	OX	GLY	P-value
N (Females)	20 (14)	16 (7)	
Age	27.7 ± 5.3	24.6 ± 6.2	0.075
BMI (kg/m^2^)	22.2 ± 2.6	22.6 ± 3.0	0.78
Type I fibers (%)	58.6 ± 5.6	29.8 ± 6.6	<0.001
Insulin sensitivity (SI_galvin_)	1.75 ± 0.70	0.77 ± 0.29	<0.001
Glucose (K_G_)	1.2 ± 0.5	1.0 ± 0.3	0.24
Plasma glucose (mM)	4.6 ± 0.4	4.7 ± 0.5	0.43
Plasma insulin (mU/L)	3.2 ± 2.4	6.4 ± 2.8	0.0002

Data are presented as means ± SD. P-values were calculated with an unpaired, two-sided Student's t-test.

Lipidomics detected 242 lipid species across 12 lipid classes, yielding a signature consistent with that observed in mouse (Figure 4B; see also Fig. 1B). Phenotypic markers corroborated the fibre-type classification: cardiolipin levels were elevated and anserine levels reduced in OX versus GLY (Figure 4C–D).

OPLS-DA identified lipids discriminating between OX and GLY, with a significant and validated model (Q^2^ = 0.36, pQ^2^ = 0.004). Compared with OX, GLY muscle had higher levels of several C18-sphingolipids, including SM(d18:1/18:0), SM(d18:1/18:1), SM(d18:0/18:0), and Cer(d18:1/18:1), Cer(d18:0/18:0), as well as SM(d18:1/24:0). In contrast, phospholipids such as PE(38:4), PC(36:2), and multiple cardiolipins (e.g., CL(72:9), CL(74:8), CL(74:11)) were enriched in OX (Fig. 4E).

To identify lipids most predictive of insulin sensitivity, we applied elastic net regression to the full lipidomics dataset. Model hyperparameters were optimized by 10-fold cross-validation, and the final model showed robust performance (mean squared error: 0.24 ± 0.11 across 1000 bootstrap resamples). Lipids were ranked by absolute regression coefficients (Fig. 4F). Positive predictors included phosphatidylcholines (PCs) such as PC(30:2), PC(38:3), and lysophosphatidylcholine LPC(18:2), consistent with their roles in membrane fluidity and insulin signaling [44]. Conversely, three ceramides, GalCer(d18:1/20:3), Cer(d18:1/18:1) and Cer(d18:1/16:0), emerged as top negative predictors, in line with their association with insulin resistance [45].

Despite clear phenotype-dependent differences in SMs and DAGs, these classes did not rank among the top predictors of insulin sensitivity, suggesting that specific ceramide species, rather than SMs or DAGs, are more closely linked to insulin sensitivity in human skeletal muscle in this cohort.

### 3.6 Fibre-type-specific sphingomyelin-ceramide cycle in human skeletal muscle

Building on the fibre-type-dependent lipidomic signatures, we next asked whether differences in ceramide content could be explained by variation in enzymes regulating ceramide metabolism, focusing on *de novo* synthesis and sphingomyelin-ceramide cycling.

**Figure 5:**
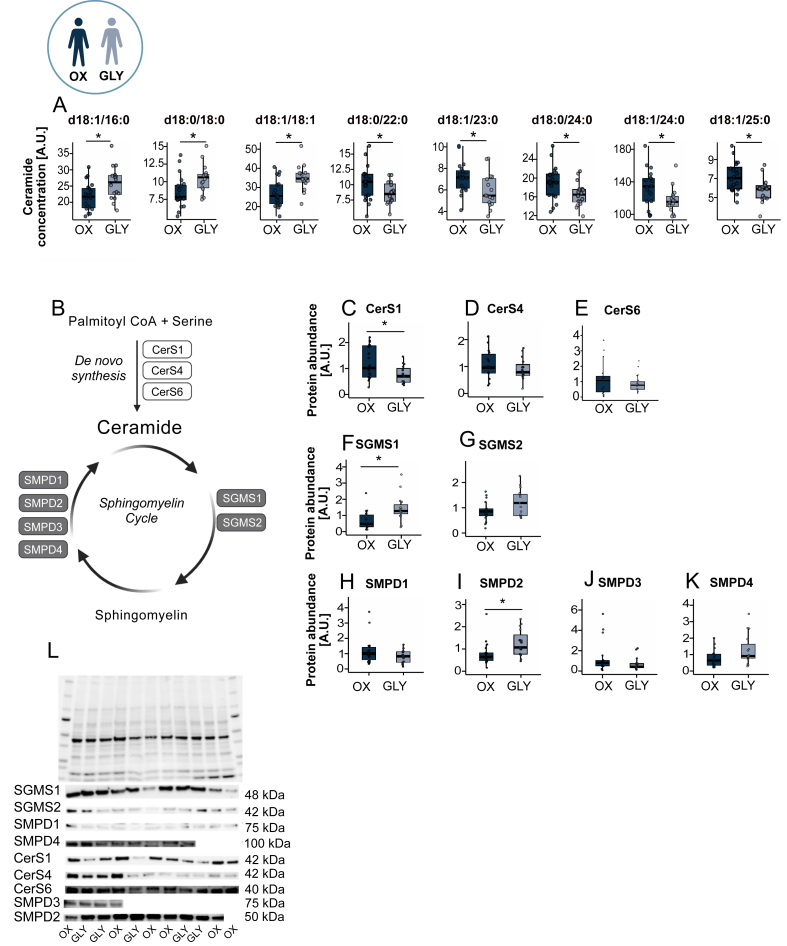
**Ceramide metabolism and acyl-chain distribution in human type I (OX) and type II-dominant (GLY) muscle phenotypes**. (a) Ceramide acyl-chain distribution in OX (n = 20) and GLY (n = 16) muscle samples. (b) Schematic overview of ceramide metabolic pathways, depicting *de novo* synthesis and the sphingomyelin–ceramide cycle. (c–e) Protein abundance of CerS1, CerS4, and CerS6 in OX (n = 20; CerS6 n = 19) and GLY (n = 16) muscle. (f–g) Protein abundance of SGMS1 and SGMS2 in OX (n = 20) and GLY (n = 16) muscle. (h–k) Protein abundance of SMPD1, SMPD2, SMPD3, and SMPD4, with sample sizes OX: n = 20 (SMPD2: n = 18; SMPD3: n = 19) and GLY: n = 16. (l) Representative Western blots, with MemCode staining used as a total-protein loading control. Box plots display the median, IQR, and ±1.5 × IQR whiskers with individual points overlaid. Statistical significance was assessed using two-sided unpaired Student's t-tests (p < 0.05). Abbreviations: OX, oxidative/type I–dominant muscle; GLY, glycolytic/type II–dominant muscle. Representative immunoblots for each protein were obtained from the same membrane.

Lipid species discriminating OX and GLY reflected distinct ceramide acyl-chain profiles consistent with fibre-type specialization. Glycolytic muscle (GLY) was enriched in long-chain ceramides [Cer(d18:1/16:0), Cer(d18:0/18:0), and Cer(d18:1/18:1), here denoted as C16- and C18-ceramides], whereas oxidative muscle (OX) showed higher very-long-chain ceramides [Cer(d18:0/22:0), Cer(d18:1/23:0), Cer(d18:0/24:0), Cer(d18:1/24:0), and Cer(d18:1/25:0), here denoted as C22-25-ceramides] (Figure 5A). These findings mirror our mouse data, supporting conserved fibre-type–dependent ceramide composition across species.

We next assessed ceramide synthases linked to these acyl-chain profiles in human muscle. Surprisingly, CerS1, the enzyme canonically linked to C18-ceramide synthesis [46], was not upregulated in GLY muscle despite its higher C18-ceramide content; instead, CerS1 was significantly higher in OX (Figure 5C,I), and CerS4 did not differ between phenotypes (Figure 5D,I). Likewise, CerS6, which preferentially generates C16-ceramides [40], tended to be higher in OX than GLY (non-significant trend, Figure 5E,I), despite higher C16-ceramide levels in GLY. Together, these findings indicate that the greater accumulation of C16–C18 ceramide in glycolytic muscle is unlikely to be driven by increased *de novo* synthesis through CerS isoforms and instead may reflect altered ceramide turnover and/or recycling (Fig. 5B).

We next evaluated SGMSs and SMPDs enzymes involved in sphingomyelin-ceramide cycling. SGMS1 expression was significantly elevated in GLY (Figure 5F,I), whereas SGMS2 was only slightly higher (p = 0.052; Figure 5G,I). Among ceramide-generating SMPDs, SMPD2 was significantly higher in GLY (Figure 5I,I), and SMPD4 showed a similar trend (p = 0.053, Figure 5K,I), while SMPD1 and SMPD3 did not differ (Figure 5H,J and I). These data support a role for altered sphingomyelin turnover, via increased SMPD-mediated hydrolysis, in ceramide accumulation in glycolytic muscle.

To complement ceramides data, we examined SM and DAG profiles. Consistent with earlier observations, GLY muscle had higher levels of several SM species, including SM(d18:1/18:0), SM(d18:1/18:1), SM(d18:1/24:0), and SM(d18:1/24:2) (Supplementary Fig. 3), whereas DAG species showed limited differences; only DAG(36:3) was significantly higher in OX (Supplementary Fig. 4).

Collectively, these findings indicate that ceramide-centered sphingolipid metabolism, rather than DAG turnover, is the dominant lipid regulatory axis linking muscle fibre-type to insulin sensitivity in humans, and highlight SGMS1, SGMS2, and SMPD2 as potential regulatory nodes mediating these effects.

## 4 Discussion

Our study shows that skeletal muscle fibre-type composition is a key determinant of ceramide metabolism, yielding distinct lipid signatures between oxidative and glycolytic phenotypes. We also implicate sphingomyelin cycling, via SGMS2-mediated ceramide clearance, as a regulator of ceramide accumulation during lipid-induced insulin resistance. These findings expand the current focus on *de novo* ceramide synthesis regulation [7], and support a broader, compartment-specific model of ceramide regulation.

Rather than total ceramide content, ceramide fatty acyl-chain composition emerged as a principal feature differentiating muscle phenotypes. Oxidative muscles (mouse soleus, human OX-dominant vastus lateralis) were enriched in very-long-chain ceramides (C22–C25), while glycolytic muscles (e.g., mouse vastus lateralis and human GLY-dominant vastus lateralis) displayed higher levels of long-chain C18-ceramides. Under high-fat diet (HFD)-induced insulin resistance, C18-ceramide levels increased across all muscle types, with the most pronounced increase in the oxidative soleus. This suggests that oxidative muscles may be especially vulnerable to ceramide dysregulation under metabolic stress, consistent with prior studies showing that oxidative fibres preferentially lose insulin sensitivity in response to lipid load as shown in humans [47] and mice [48]. However, these interpretations are based on total tissue-level measurements which may obscure changes in specific subcellular pools.

The C18-ceramide rise in soleus occurred without a corresponding change in CerS1, the enzyme responsible for their *de novo* synthesis [7]. Consistent with altered cycling, SGMS2 was selectively downregulated. Since SGMS2 catalyzes sphingomyelin synthesis with concomitant production of 1,2-diacylglycerol (DAG) [49], its downregulation could potentially shift the balance toward increased ceramide and reduced sphingomyelin levels. Such shifts have been linked to insulin resistance and altered membrane organization [50,51]. However, whether the magnitude of SGMS2 downregulation observed here is sufficient to drive these effects, or how alterations to ceramide and sphingomyelin cycling translate to lipid raft remodeling in vivo remains unclear.

Our data are consistent with previous work on SGMS1 and 2 [42,52] suggesting that relative downregulation of SGMS2, rather than changes in SGMS1, may contribute to C18-ceramide accumulation and insulin resistance in oxidative muscle under lipid overload. Previous studies show that exercise selectively increases SGMS1 expression and enhances mitochondrial function without altering SGMS2 or total ceramide content [30], and rodent studies likewise implicate SGMS1 in maintaining mitochondrial integrity via ceramide-ROS signaling [53]. Conversely, inhibition of SGMS2 has been reported to impair insulin-stimulated glycogen synthesis and attenuate Akt and GSK3 phosphorylation [42]. Taken together, these findings suggest that SGMS2 contributes to ceramide accumulation as part of a broader regulatory network rather than acting as a sole driver.

The divergent effects of the SGMS isoforms likely reflect their distinct sub-cellular localizations: SGMS1 (Golgi/ER) may regulate mitochondria-associated ceramides, whereas SGMS2 (plasma membrane) promotes ceramide-rich platform formation that disrupts membrane organisation and blunts insulin signaling [54]. Whole-body SGMS2 knockout mice even show paradoxical improvements in glucose clearance [52], suggesting compensatory or tissue-specific adaptations that can mask its intrinsic role in muscle. Our siRNA experiments reinforce this view. Knockdown of SGMS2 in primary human myoblasts produced a broader increase in ceramide species than SGMS1 silencing, but these effects were moderate; hence, we interpret SGMS2 as a contributing node in ceramide homeostasis. This supports a model where ceramide accumulation is governed by both *de novo* synthesis and organelle- or membrane proximal ceramide pools, with SGMS2 likely influencing plasma membrane ceramide level, a pool closely linked to impaired insulin signaling.

In parallel, enzyme mapping in mouse muscles confirmed fibre-type specific ceramide metabolism: CerS1 and C18-ceramides were enriched in glycolytic muscle, while CerS4 and very-long-chain ceramides (C22–C24) were elevated in the oxidative soleus muscle. Under HFD, C18-ceramide increased across muscles, most prominently in soleus, whereas very-long-chain species declined selectively in the glycolytic vastus lateralis. Again, interpretation should consider that these observations reflect total tissue contents.

Sphingomyelin-ceramide cycling enzymes also followed phenotype-specific pattern. SGMS2 and SMPD4 levels, implicated in plasma membrane ceramide turnover [41] were higher in glycolytic muscle, while SGMS1 and SMPD1, linked to lysosomal ceramide pools [41,55], were enriched in oxidative muscle. This suggests that not only ceramide composition, but its subcellular location varies by muscle phenotype. Beyond sphingomyelin cycling, flux into glycosphingolipid and ceramide-1-phosphate pathways could differ by phenotype and modulate apparent ceramide levels, which we could not quantify here. These findings align with previous reports in animal models that loss of very-long-chain ceramides (i.e., C22–C24) worsens metabolic function in mice [56], while loss of C18-ceramides protects against HFD-induced insulin resistance [7]. Our study adds a critical nuance: these ceramide species show divergent effects depending on muscle fibre-type context, a factor rarely accounted for in prior research.

Extending these insights to humans, we found that glycolytic muscle (GLY) accumulates more C18-ceramide levels and fewer very-long-chain ceramides (C22–C24) compared to oxidative muscle (OX), mirroring the mouse data. As individuals with more oxidative fibres are frequently reported as being more insulin sensitive [13,26], this raises the question of whether ceramide composition or muscle phenotype itself underlies this association. To disentangle this, we applied elastic net regression, which accounts for multicollinearity while selecting relevant predictors. Long-chained ceramides, particularly Cer(d18:1/18:1) and Cer(d18:1/16:0), emerged as strong negative predictors of insulin sensitivity, supporting their functional relevance. These results indicate that C18 ceramides are associated with insulin resistance independent of BMI and total skeletal muscle lipid content.

Interestingly, higher levels of C18-ceramides and C16-ceramide in GLY muscle under basal conditions occurred despite reduced CerS1 levels and no difference in CerS6 expression, which is compatible with involvement of the sphingomyelin-ceramide cycle and/or other complex sphingolipid pathways returning to ceramide. These data support a model in which ceramide homeostasis is regulated both by *de novo* synthesis and turnover/clearance, with their relative contribution dictated by fibre type. For example, as observed in mouse, oxidative muscles may limit ceramide accumulation via low CerS1 output and comparatively higher SGMS1-mediated clearance, whereas glycolytic muscles may accumulate ceramides through elevated SMPD and reduced SGMS1-dependent clearance.

Recognising this fibre-type and compartment-specific complexity is critical to resolving conflicting findings in the field. Several interventions have improved insulin sensitivity without altering total muscle ceramide content [57], possibly due to shifts in specific ceramide subspecies or subcellular pools [58]. In addition, differential flux into glycosphingolipids and ceramide-1-phosphate may unmask meaningful changes in ceramide metabolism when only total content is assessed. Our data indicates that total ceramide levels are not informative on their own: functional insights require species-resolved and spatially resolved lipidomics ideally complemented by pathway-level flux measurements (e.g., stable isotope tracing) to capture turnover into and out of complex sphingolipids.

Several limitations should be acknowledged. First, we lacked subcellular resolution of ceramide localization, as all measurements reflect total tissue lipid levels. Future investigations using organelle-targeted lipidomics or imaging mass spectrometry are therefore warranted. Second, we did not quantify flux into glycosphingolipids or ceramide-1-phosphate, nor did we assess enzymes involved in glycosphingolipid metabolism, which limits insight into downstream ceramide fate when inferring the specific contribution of SGMS2. Third, we did not assess the role of serine palmiotyltransferase (SPTLC1/2) in the *de novo* ceramide synthesis. Differences at this initial step of ceramide production could contribute to fibre-type differences in ceramide levels. Although SPT activity was not measured in the present study, systemic inhibition of SPT (for example, with myriocin) is known to reduce skeletal muscle ceramide content and improve insulin sensitivity in rodent models [6]. Future studies using SPT inhibitors could help delineate the contribution of *de novo* synthesis to the muscle-specific ceramide profiles observed here. Fourth, we did not directly assess the impact of SGMS2 modulation on insulin signaling, and SGMS1/2 silencing experiments were performed in human myoblasts under basal conditions. Future studies should extend these experiments to differentiated myotubes and include palmitate exposure to better model obesity-associated alterations in ceramide metabolism and insulin resistance. Also, while human participants were stratified by skeletal muscle phenotype, residual lifestyle confounders may persist. However, previous data [13] indicate that differences in physical activity do not account for the observed variations in insulin sensitivity. Finally, sex differences could not be concluded due to the limited sample size, despite their known relevance to muscle lipid metabolism. Therefore, future studies should explicitly address sex as a biological variable.

In summary, our findings demonstrate that muscle fibre-type dictates sphingolipid metabolism through distinct regulatory pathways, with evidence compatible with SGMS2-mediated sphingomyelin-ceramide cycling as one contributing mechanism among several (including turnover into other complex sphingolipids). This fibre-type-specificity governs ceramide composition and its adaptation to high-fat diet-induced metabolic stress, providing new mechanistic insight into the lipid basis of insulin resistance. Interventions that consider ceramide turnover beyond biosynthesis alone and target specific subspecies and subcellular pools may hold promise in preserving muscle insulin sensitivity, especially in oxidative muscle phenotypes.

## CRediT authorship contribution statement

**Tova Eurén:** Writing – review & editing, Writing – original draft, Visualization, Formal analysis, Data curation. **Mikael Flockhart:** Writing – review & editing, Formal analysis, Data curation. **Timotej Strmeň:** Data curation. **Xin Zhou:** Data curation. **Oscar Horwath:** Data curation. **William Apró:** Project administration, Data curation. **Sarah J. Blackwood:** Data curation. **Dominik Tischer:** Data curation. **Marcus Moberg:** Data curation. **Pär Steneberg:** Writing – review & editing, Data curation. **Helena Edlund:** Writing – review & editing, Data curation. **Abram Katz:** Writing – review & editing, Funding acquisition, Conceptualization. **Elin Chorell:** Writing – review & editing, Writing – original draft, Supervision, Resources, Project administration, Funding acquisition, Conceptualization.

## Declaration of generative AI and AI-assisted technologies in the manuscript preparation process

During the preparation of this work the author(s) used OpenAI's GPT-5 to assist with language editing. After using this tool, the author(s) reviewed and edited the content as needed and take(s) full responsibility for the content of the published article.

## Funding

This work was supported by 10.13039/501100004359Swedish Research Council (2021-01091), Swedish Diabetes Foundation (DIA2022-726), and Åke Wibergs Stiftelse (M22-0057).

## Declaration of competing interest

The authors declare that they have no known competing financial interests or personal relationships that could have appeared to influence the work reported in this paper.

## Data Availability

All processed mass-spectrometry data generated in this study are available at Zenodo Repository: 10.5281/zenodo.15730840. Other data availible upon request.
