# Supplementary material for: Ceramide metabolism in oxidative and glycolytic muscle: Significance for lipid-induced insulin resistance
Source: Mol Metab. 2026 Feb 16;106:102336. doi: 10.1016/j.molmet.2026.102336 (PMC12955587; doi:10.1016/j.molmet.2026.102336)
Supplement: Multimedia component 1 [file mmc1.pdf]

Supplementary Materials

Table of Contents

Supplementary Table 1.
List of primary and secondary antibodies used for protein measurements.

Supplementary Figure 1.
Sphingomyelin acyl-chain distribution in oxidative soleus, intermediate EDL, and glycolytic vastus lateralis skeletal muscles of mice.

Supplementary Figure 2.
Diacylglycerol acyl-chain distribution in oxidative soleus, intermediate EDL, and glycolytic vastus lateralis skeletal muscles of mice.

Supplementary Figure 3.
Sphingomyelin acyl-chain distribution in type I (OX) and type II-dominant (GLY) human muscle phenotypes.

Supplementary Figure 4.
Diacylglycerol acyl-chain distribution in type I (OX) and type II-dominant (GLY) human muscle phenotypes.

Supplementary Figure 5.
Intraperitoneal glucose tolerance and glucose-stimulated insulin secretion in control and high-fat diet–fed mice.

Table S1: List of primary and secondary antibodies used for protein measurements.

Target protein	Data	Supplier	Catalog ID
**SGMS1**	Mouse	Proteintech	#19050-1-AP
**SGMS2**	Mouse	Thermo Fisher Scientific	#PA5-26744
**SMPD1**	Mouse	Proteintech	#14609-1-AP
**SMPD4**	Mouse	Thermo Fisher Scientific	#PA5-110401
**CerS1**	Mouse	Sigma-Aldrich	#SAB2104843
**CerS4**	Mouse	Thermo Fisher Scientific	#PA5-110401
**SGMS1**	Human Myoblasts	Abcam	#ab235057
**SGMS2**	Human Myoblasts	Abcam	#ab237681
**β-Actin**	Human Myoblasts	Cell signaling	#4967

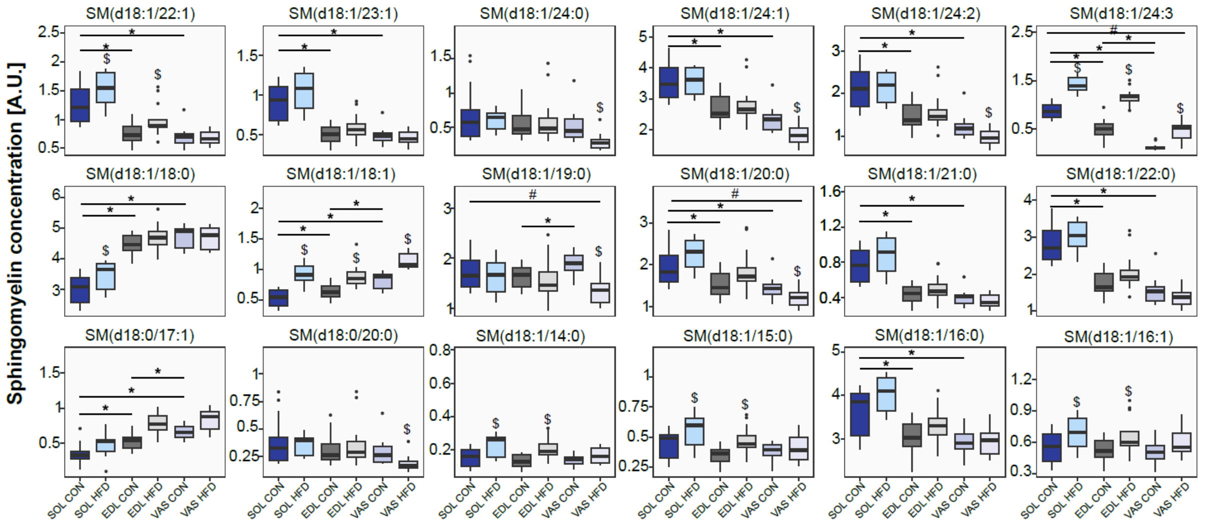

Supplementary Figure 1. Sphingomyelinacyl-chain distribution in oxidative soleus, intermediate EDL, and glycolytic vastus lateralis skeletal muscles of mice.

Sphingomyelin species concentrations are shown for soleus, extensor digitorum longus, and vastus lateralis (n=12per muscle per treatment).

Box plots show the median, IQR, and ±1.5×IQRwhiskers. Sphingomyelin concentrations were normalized using a labelled internal standard, and log_2-transformed values were analysed using two-way ANOVA (muscle × treatment) with pairwise two-sided Student's t-tests. Significance symbols: # interaction effect; * main effect of muscle; $ main effect of treatment; statistical significance set at $p < 0.05$. Abbreviations: SOL, soleus; EDL, extensor digitorum longus; VAS, vastus lateralis. CON, control diet; HFD, high-fat diet.

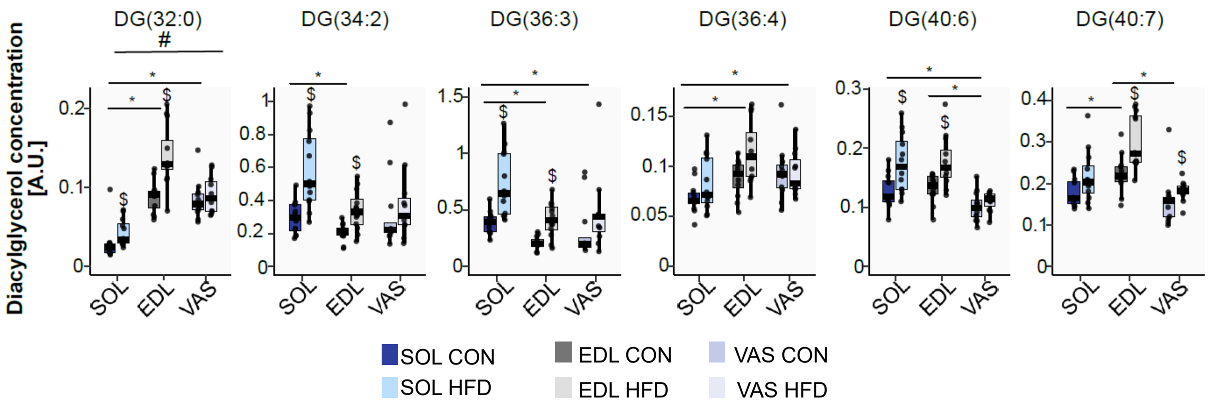

Supplementary Figure2. Diacylglycerol acyl-chain distribution in oxidative soleus, intermediate EDL, and glycolytic vastus lateralis skeletal muscles of mice.

Diacylglycerol species concentrations are shown for soleus, extensor digitorum longus, and vastus lateralis (n=12 per muscle per treatment). Box plots show the median, IQR, and ±1.5×IQR whiskers with individual data points overlaid. Diacylglycerol concentrations were normalized using a labelled internal standard, and log_2-transformed values were analysed by two-way ANOVA (muscle × treatment) with pairwise two-sided Student's t-tests. Significance symbols: # interaction effect; * main effect of muscle; $ main effect of treatment; statistical significance set at $p < 0.05$. Abbreviations: SOL, soleus; EDL, extensor digitorum longus; VAS, vastus lateralis. CON, control diet; HFD, high-fat diet.

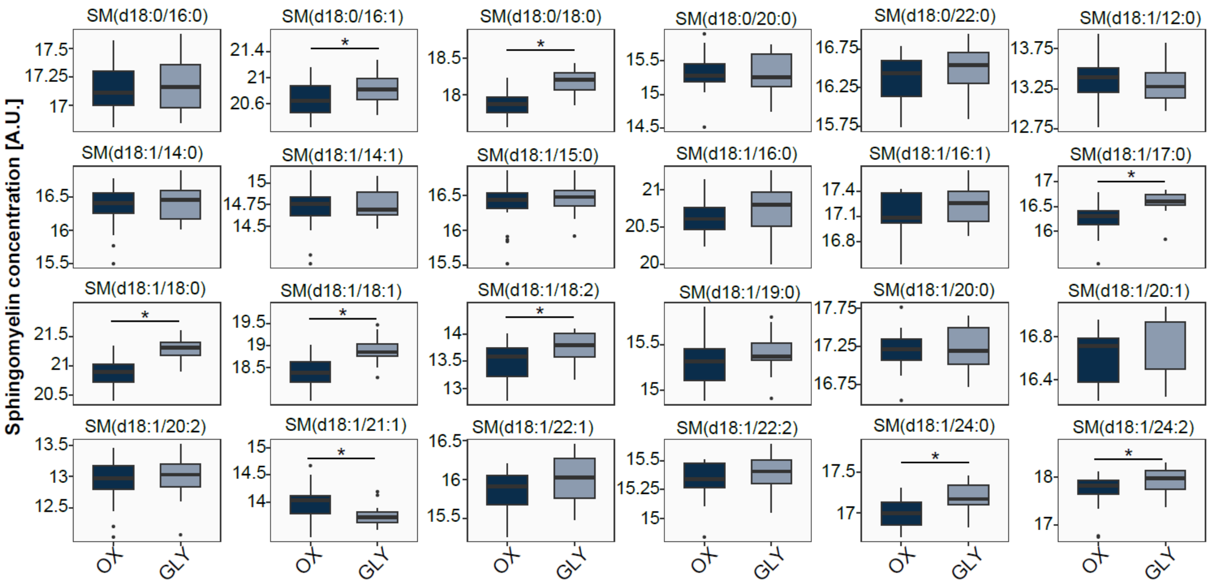

Supplementary Figure 3. Sphingomyelin acyl-chain distribution in type I (OX) and type II-dominant (GLY) human muscle phenotypes.

Sphingomyelin species concentrations are shown for type I–dominant (OX; n=20) and type II–dominant (GLY; n=16) muscle. Box plots show the median, IQR, and ±1.5×IQR whiskers. Statistical significance was assessed using two-sided Student's t-tests ($p < 0.05$). Abbreviations: OX, oxidative/type I–dominant muscle; GLY, glycolytic/type II–dominant muscle.

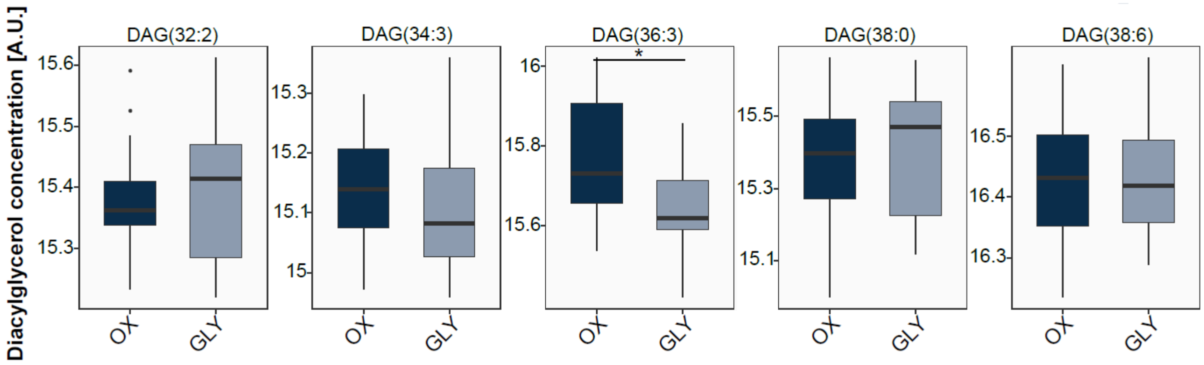

Supplementary Figure 4. Diacylglycerol acyl-chain distribution in type I (OX) and type II-dominant (GLY) human muscle phenotypes.

Diacylglycerol species concentrations are shown for type I–dominant (OX; n=20) and type II–dominant (GLY; n=16) muscle. Box plots show the median, IQR, and ±1.5×IQR whiskers with individual data points overlaid. Statistical significance was assessed using two-sided Student's t-tests ($p < 0.05$). Abbreviations: OX, oxidative/type I–dominant muscle; GLY, glycolytic/type II–dominant muscle.

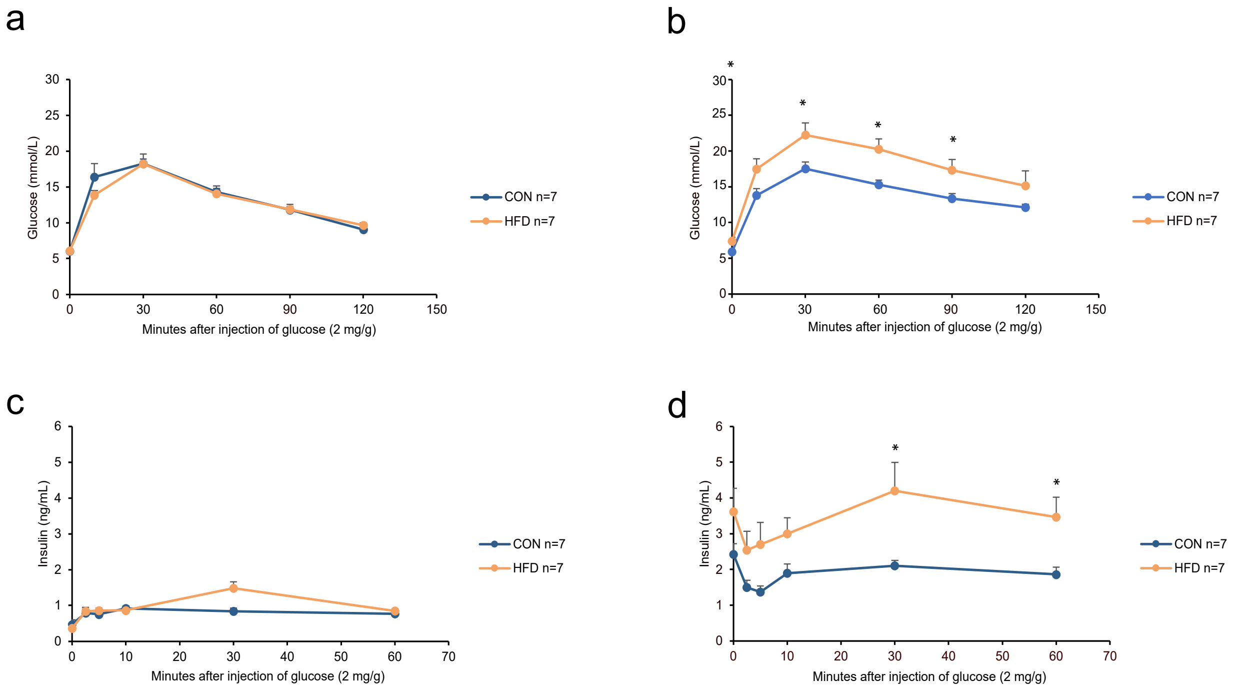

Supplementary Figure 5. Intraperitoneal glucose tolerance and glucose-stimulated insulin secretion in control and high-fat diet–fed mice.

(a) IPGTT at baseline (start of diet) in control (CON, n=7) and high-fat diet (HFD, n=7) mice.
(b) IPGTT after 9 weeks of diet intervention in CON (n=7) and HFD (n=7) mice.
(c) IPGSIS at baseline (start of diet) in CON (n=7) and HFD (n=7) mice.
(d) IPGSIS after 9 weeks of diet intervention in CON (n=7) and HFD (n=7) mice.

Data are presented as mean ± SEM. Statistical significance was assessed using two-sided unpaired Student's t-tests at each time point ($p < 0.05$ was considered significant (*)).

Abbreviations: IPGTT, intraperitoneal glucose tolerance test; IPGSIS, intraperitoneal glucose-stimulated insulin secretion; CON, control diet; HFD, high-fat diet.
